# Proteomic profiling of exosomes leads to the identification of a candidate biomarker for prostate cancer progression

**DOI:** 10.1016/j.gendis.2024.101463

**Published:** 2024-11-12

**Authors:** MyeongHoon Yeon, Ah Reum Lee, Youngbum Yoo, Won-Kyeong Kim, Hyeon-Bin Shin, Hae Un Kook, Soon-Cheol Ahn, Myunggon Ko, Inkyung Jung, Chan Young Park, Young-Kyo Seo

**Affiliations:** a Aging Convergence Research Center, Korea Research Institute of Bioscience and Biotechnology (KRIBB), Daejeon 34141, Republic of Korea; b Department of Biomedical Engineering, Ulsan National University of Science and Technology (UNIST), Ulsan 44919, South Korea; c Metabolic Regulation Research Center, Korea Research Institute of Bioscience and Biotechnology (KRIBB), Daejeon 34141, Republic of Korea; d Department of Biomolecular Science, KRIBB School of Bioscience, Korea University of Science and Technology (UST), Daejeon 34141, Republic of Korea; e Department of Microbiology and Immunology, Pusan National University School of Medicine, Yangsan 50612, South Korea; f Department of Biological Sciences, Ulsan National Institute of Science and Technology (UNIST), Ulsan, South Korea

Extracting exosomes from bodily fluids offers a promising approach to overcoming inherent limitations, enabling the identification of potential biomarkers, especially those present in low abundance.^1^ PC-3 cells, known for their high metastatic potential compared with LNCaP cells, are widely used as a model for prostate cancer progression.^2^^,^^3^ However, the mechanisms underlying their metastatic characteristics remain unclear. Therefore, there is a critical need to investigate diagnostic methods for prostate cancer, as current techniques have various limitations that can lead to overtreatment due to their lack of precision.^3^ This study isolated exosomes from PC-3 and LNCaP cells through sequential ultracentrifugation to analyze their proteomic profiles using nano-liquid chromatography coupled to tandem mass spectrometry. Western blotting and quantitative reverse transcription-PCR validation revealed a higher abundance of CD146 (MCAM) in PC-3 exosomes, indicating that aggressive prostate cancer exhibits elevated levels of adhesion or cohesion proteins.^4^ We demonstrated that knocking out CD146 in PC-3 cells via the CRISPR-Cas9 system inhibited cell proliferation and invasion. Overall, CD146 was strongly associated with PC-3 cell mobility *in vitro*, suggesting that CD146 is a valuable candidate biomarker for prostate cancer progression.

To determine the effect of exosomes on prostate cancer cell motility, proliferation potential tests were executed using an exosome enrichment medium specifically tailored for PC-3 cells and LNCap cells, respectively (Supplementary Movie). We found that exosome enrichment medium-treated PC-3 cells moved significantly faster with more directional motility than LNCap cells did (Fig. 1A). Proliferation of the exosome enrichment medium-treated cells showed that numbers of PC-3 cells were much higher than those of LNcaP cells in 24 h (Fig. 1B). The exosomes from two human prostate cancer cell lines, LNCaP and PC-3 were collected and purified based on differential ultracentrifugation from the culture supernatants (Fig. S1). Transmission electron micrographs revealed that the isolated exosome particles consisted of primarily round-shaped vesicles (Fig. 1C). The images indicate that both exosome vesicles from LNCaP and PC-3 cells exhibit relatively similar sizes with diameters ranging from 80 to 250 nm. The different categories of exosomal marker proteins, HSP70,^5^ cytosolic protein, and LAMP-1, the transmembrane molecule, were confirmed via western blotting (Fig. 1D). To profile the protein contents from the two types of exosome vesicles, the vesicles were treated with acetone to precipitate their protein contents. The purified proteins were visualized on an SDS-PAGE gel by silver stain and at least 8 bands (Fig. S2) were identified. The protein assessment of the exosome preparations indicated quite similar amounts of vesicles for both LNCaP (19.55 ± 1.24 μg/mL) and PC-3 cells (18.38 ± 2.16 μg/mL). A quantity of 10 μg of total protein was loaded onto a 10% SDS-PAGE gel, and the subsequent analysis was conducted with liquid chromatography coupled to tandem mass spectrometry (Fig. S3). Scaffold (version Scaffold_4.8.7, Proteome Software Inc., Portland, OR) was used to validate the peptide and protein identifications. Protein identifications were accepted when they could be established at greater than 99.0% probability and contained at least 3 identified peptides of 397 non-redundant proteins, and 154 (24%) were identified via both fractionation platforms (Table S1). Differential protein expression analysis revealed that exosomes from LNCaP and PC-3 cells contain distinct protein profiles. Among the identified proteins, 105 were significantly up-regulated in PC-3 exosomes versus LNCaP exosomes. To further investigate these findings, we focused on the top 20 most up-regulated proteins and manually performed quantitative reverse transcription-PCR using RNA extracted from the cells (Fig. 1F and Table S2). Given that CD146 (MCAM) was consistently up-regulated in both experimental approaches, we decided to further study its role and significance in prostate cancer. Proteomic analysis revealed that CD146 expression was approximately 8-fold higher in PC-3 cells than in LNCap cells (Fig. 1G). Regarding the strong metastatic potential of PC-3 cells, the CD146 was regarded to play a role in cell-to-cell adhesion at intercellular junctions. The exosomal proteins isolated from LNCaP and PC-3 cells were subjected to Western blot analysis. CD146 was markedly elevated in exosomes from the highly metastatic PC-3 cells versus the LNCaP cells (Fig. 1H). Notably, the exosomal markers CD9 and CD63 were also detected to confirm the purity of exosome preparations. We performed gene knockout using CRISPR/Cas9 systems to investigate the effect of CD146 on cancer metastasis in PC-3 cells. The absence of CD146 expression was confirmed in exosomes isolated from CD146 knockout PC-3 cells (Fig. 1I). The proliferation rate of CD146 knockout cell lines was observed using MTT assay. The proliferative activity of PC-3 cells exhibited a progressive increase over 24, 48, and 72 h, with a notable reduction observed in CD146 knockout conditions, indicating a significant impact on the proliferation of PC-3 cells (Fig. 1J). Moreover, to elucidate the role of CD146 in regulating the proliferative dynamics of PC3 cells, we conducted a comparative analysis of colony formation. After a 7-day culture, CD146 knockout PC3 cells exhibited a significant reduction in colony-forming ability compared with the wild-type cells (Fig. 1K). Quantitative analysis revealed a notable decrease in the size of colonies formed by CD146 knockout cells, indicating a compromised ability to sustain larger clonal growth in the absence of CD146 (Fig. 1L). Next, we examined whether CD146 knockout affected cell invasion and migration. We examined the cell migration of CD146 knockout cells using the wound healing assay. Equal numbers of cells were plated, and the cell monolayer was scraped and monitored for wound closure. After 24 h, the wound gaps were quantified for statistical analysis. While control cells migrated into the wound, the cell migration of CD146 knockout cells was reduced significantly (Fig. 1M, N). Moreover, to verify the role of CD146 in prostate cancer progression, we conducted rescue experiments and found that reintroducing CD146 into CD146 knockout PC-3 cells restored both cell proliferation (Fig. 1O) and migration (Fig. 1P, Q), confirming its essential role in these processes. Given the *in vitro* activity of CD146^−/−^ PC-3 against wound healing migration, we validated the potential effects of CD146 *in vivo*. Immunohistochemical analyses were performed to evaluate CD146 expression in prostate adenocarcinoma tissue (Fig. 1R). The expression patterns of CD146 in prostate adenocarcinoma tissues were significantly higher than those in the normal controls (Fig. 1S). Notably, the tissue with a Gleason score of 9 demonstrated significantly higher CD146 expression compared with the sample with a Gleason score of 6 (Fig. S4). These results strongly suggest that CD146 might be required for the growth of prostate cancer PC-3 cells.

**Figure 1:**
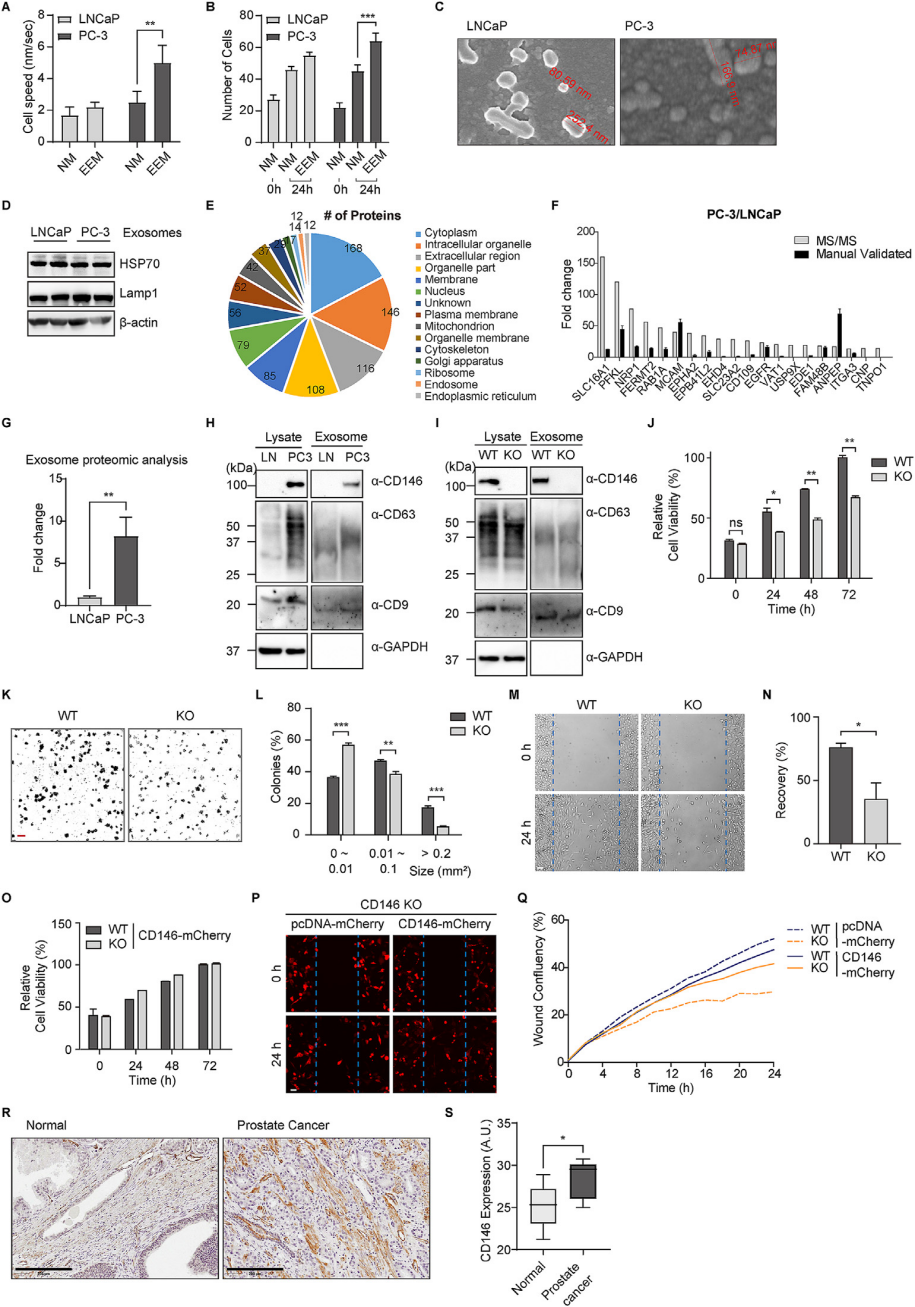
Proteomic profiling of exosomes leads to the identification of a candidate biomarker for prostate cancer progression. **(A)** Average PC-3 and LNCaP cell speeds were measured. **(B)** The proliferation assay demonstrated a significant dose-dependent increase in PC-3 and LNCaP cells with the addition of exosome enrichment medium (EEM) and without EEM after 24 h. **(C)** Transmission electron microscope (TEM) images of exosomes derived from PC-3 versus LNCaP cells. **(D)** Validation of representative exosome proteins. The selected proteins HSP70 and Lamp1 were tested as positive controls. **(E)** The distribution of proteins identified from Gene Ontology Cellular Component analysis. **(F)** Quantitative reverse transcription-PCR results of 20 up-regulated genes. **(G)** Comparison of CD146 in LNCap and PC-3 exosomes through proteomics analysis. **(H)** Western blot analysis of exosomes isolated from LNCaP and PC-3 cells. CD9 and CD63 were used as exosome markers. GAPDH was used as an endogenous control. **(I)** Western blot analysis of exosomes isolated from CD146 wild-type (WT) and knockout (KO) PC-3 cells. CD9 and CD63 were used as exosome markers. GAPDH was used as an endogenous control. **(J)** Cell viability was determined with MTT assay at 24, 48, and 72 h in PC-3 cells. **(K)** Clonogenic assay of CD146 WT and KO cells. Scale bar, 1 mm. **(L)** Quantification of the number of colonies. **(M)** Representative images of the areas covered by PC-3 WT or CD146 KO cells 0 and 24 h after wounding. Scale bar, 50 μm. **(N)** The wound area was calculated using Image J and normalized to the size of the original wound (Time 0). PC-3 and *CD146*^*−/−*^ PC-3 cells resuspended of PBS were mixed and subcutaneously injected in 6-to-8-week-old male NOD/SCID mice. **(O)** Cell viability was determined with MTT assay at 24, 48, and 72 h in CD146 WT and KO PC-3 cells expressing CD146-mCherry. **(P)** Representative images of the areas covered by PC-3 WT or CD146 KO cells expressing mCherry or CD146-mCherry 0 and 24 h after wounding. Scale bar, 50 μm. **(Q)** The wound area was calculated by the integrated metric relative wound density part of the live content cell imaging system Incucyte HD and normalized to the size of the original wound (Time 0). The data were presented as mean ± standard error; *n* = 3; ∗∗∗*P* < 0.001; one-way ANOVA was performed for Dunnett's multiple comparison test. **(R)** Immunohistochemistry showed CD146 expression in prostate adenocarcinoma tissue. Scale bar: 200 μm. **(S)** The total optical density (OD) in DAB-stained sections was quantified using the automated OD measurement algorithm within the QuPath software. The mean OD values were subsequently calculated. Cell counts ranged from 10,000 to 20,000 per tissue section. The data were presented as mean ± standard deviation; *N* = 10; ∗*P* < 0.05; unpaired *t*-test.

In conclusion, these data suggest that CD146, a new protein, could play a role in the high adhesion of PC-3 to other tissues and could offer the potential to develop a new diagnostic marker for prostate cancer progression. Our observations shown here should shed more light on the precise mechanisms involved in prostate tumor metastasis. CD146 will need to be studied further to assess its potential as a target for the treatment of prostate cancer.

## CRediT authorship contribution statement

**MyeongHoon Yeon:** Data curation, Formal analysis, Software, Validation. **Ah Reum Lee:** Data curation, Formal analysis, Validation, Writing – original draft. **Youngbum Yoo:** Data curation, Formal analysis. **Won-Kyeong Kim:** Data curation, Formal analysis. **Hyeon-Bin Shin:** Data curation, Formal analysis. **Hae Un Kook:** Data curation, Formal analysis. **Soon-Cheol Ahn:** Formal analysis, Validation. **Myunggon Ko:** Conceptualization, Data curation. **Inkyung Jung:** Data curation, Formal analysis. **Chan Young Park:** Data curation, Formal analysis, Visualization, Writing – original draft. **Young-Kyo Seo:** Conceptualization, Funding acquisition, Investigation, Project administration, Writing – original draft.

## Ethics declaration

All animal studies were approved by the Animal Care and Use Committee of the Korea Research Institute of Bioscience and Biotechnology (KRIBB).

## Conflict of interests

No conflict of interests for any of the authors.

## Funding

This research was supported by grants from the National Research Foundation of Korea (NRF) (No. 2022R1A2C109179013, RS-2024-00451519, RS-2024-00359310, 2018R1A5A1024340), the National Research Council of Science & Technology (NST) Aging Convergence Research Center (Korea) (No. CRC22012-200), and the Ministry of Health and Welfare of Korea (No. HV22C012800).
