# Supplementary material for: Proteomic profiling of exosomes leads to the identification of a candidate biomarker for prostate cancer progression
Source: Genes Dis. 2024 Nov 12;12(4):101463. doi: 10.1016/j.gendis.2024.101463 (PMC11995069; doi:10.1016/j.gendis.2024.101463)
Supplement: Multimedia component 1 [file mmc1.docx]

**Supplemental Material**

**Materials and Methods**

**Materials**

The goat-anti-mouse IgG and goat-anti-rabbit secondary antibody were purchase from Enzo Life Science (Farmingdale, NY, USA). The FITC-conjugated anti-rabbit IgG secondary antibody was purchased from BioFX Laboratories (Owings Mills, MD, USA). All antibodies were purchase as follows; HSP70, Lamp1, GAPDH (proteintech, 60004-1-Ig), β-actin (proteintech, 66009-1-Ig), FLAG (Sigma-Aldrich, F1804), GFP (MBL, 598), CD146 (abcam, ab75769), CD63 (proteintech, 25682-1-AP), CD9 (proteintech, 20597-1-AP), Hoechst 33342 (Invitrogen, H3570).

**Cell culture and isolation of exosomes**

Human prostate cancer cells lines, PC-3 and LNCaP were obtained from the American Type Culture Collection (ATCC, Manassas, VA, USA). These cells were maintained and cultured in RPMI1640 (BioWhiteker, Walkersville, MD) supplemented with 10% fetal bovine serum (FBS) (BioWhiteker, Walkersville, MD), 100 units/ml of penicillin and 100 μg/ml of streptomycin (BioWhiteker, Walkersville, MD) at 37°C in a humidified atmosphere with 5% CO_2_. The PC-3 cell line was cultured in Dulbecco’s modified Eagle’s medium-Ham’s F-12 medium (Lonza), supplemented with multiple additives as described by Marques ^1^. For the isolation of exosome vesicles from the two prostate cancer cell lines, culture supernatants in serum deprived DMEM media (original cell density 1×10^6^ cells/ml) were harvested. Then the exosomes were isolated as described previously with minor modifications ^2;3^. After reaching over 80% confluence, the cells were incubated with 25 ml serum free medium. After 48 h, the supernatant was collected and subjected to centrifugation steps of 4000 x g (10 min), 3,000 x g (20 min), and 10,000 x g (30 min) to remove cellular debris. Exosomes were then pelleted at 64,000 x g (110 min) for 1 h. Flow chart for the exosome purification procedure based on differential ultracentrifugation is shown in Fig S1. The speed and the length of each centrifugation step are indicated at the central line of the arrows. After each the first step, pellet is discarded, and the supernatant is kept for the next step. Total amount and concentration of exosomal proteins of the pooled samples was measured with a BCA-assay (Pierce, Rock ford, IL, USA).

**Transmission electron microscopy (TEM)**

EM imaging of vesicle preparations was performed as previously described ^3^, with some modifications.

Briefly, vesicles (about 2 μg protein) were fixed in 1% glutaraldehyde and then layered and dried on formvar coated 200 mesh copper grids (Polysciences, Inc. PA, USA). Grids were then stained 1% uranylacetate in water. Imaging took place at an accelerated voltage of 200 kV using a Tecnai G2 F30 TWIN, which is a 300 kV/FEG Transmission Electron Microscope.

**Protein analysis using LC-MS/MS**

The exosome-like vesicles (n = 3/exosome type) were resuspended in 100 μl of PBS, 2 μl triton X-100, and 5 μl phenylmethylsulfonyl fluoride with vortexing to dissolve the vesicles. The insoluble fraction was pelleted by centrifugation 20,000 g. The insoluble fraction was acetone precipitated at −20°C and digested in-gel with 200 ng modified trypsin (sequencing grade, Promega) for 18 hours at 37°C. Resulting peptides were analyzed by LC-MS/MS on an Orbitrap-XL mass spectrometer (Thermo Scientific, Waltham MA). The resulting tryptic peptides were analyzed by LC-MS/MS. All mass analyses were performed on a LTQ/Orbitrap (Thermo, Bremen, Germany) equipped with a nanoelectrospray ion source. To separate the peptide mixture we used a C18 reverse phase HPLC column (150 mm 3 75 mm i.d.) using an acetonitrile/0.1% formic acid gradient of 66 min at a flow rate of 300 nl/min. For MS/MS analysis the precursor ion scan MS spectra (m/z 400– 2000) were acquired in the Orbitrap at a resolution of 60,000 at m/z 400 with an internal lock mass. The three most intensive ions were isolated and fragmented in the linear ion trap by collisionally induced dissociation (CID). All MS/MS spectra were analyzed by sequest against the curated version of B. bacteriovorus protein sequence database (3583 entries, UniProt (http://www.uniprot.org/)) containing all the protein-coding sequences of Bdellovibrio bacteriovorus strain (ATCC 15356, DSM 50701, NCIB 9529 and HD100) and frequently observed contaminants. Carbamidomethyl cysteine and oxidized methionine were set as a fixed modification and variable modification, respectively. A maximum of two missed cleavages were allowed. The mass tolerances allowed forth MS peaks were 10 ppm, and for the MS/MS peaks they were 0.8 Da. The search results were further filtered with the following options: minimum number of matching peptides was 2 and the false discovery rate was set to 0.01.

**Western blot analysis**

Cell extracts were prepared by incubating the cells in lysis buffer [150 mM NaCl, 10 mM Tris (pH 7.4), 5 mM EDTA (pH 8.0), 1% Triton X-100, 1 mM PMSF, 20 lg/ml aprotinin, 50 lg/ml leupeptin, 1 mM benzidine, 1 mg/ml pepstatin, 8 mM sodium pyrophosphate and 20 mM β-glycerophosphate]. Forty micrograms of proteins was electrophoretically separated using sodium dodecyl sulfate–polyacrylamide gel electrophoresis (SDS–PAGE) on 8–15% gel and transferred to a [Nitrocellulose](http://www.lifetechnologies.com/kr/ko/home/life-science/protein-biology/protein-assays-analysis/western-blotting/transfer-proteins-western-blot/membranes-transfer-buffers-western-blotting/membranes-western-blotting/nitrocellulose-membranes-western-blotting.html) (NC) membrane. After blocking with TBS-T buffer [20 mM Tris (pH 7.4), 150 mM NaCl, 0.1% Tween 20] containing 5% skim milk, membranes were incubated with primary and secondary antibodies. The membranes were then washed with TBS-T buffer and visualized with ECL western blot analysis detection reagents. The density of each band was determined with a fluorescence scanner (LAS 3000, Fuji Film, Tokyo, Japan) and analyzed with Multi Gauge V3.0 software (Fuji Film).

**Immunocytochemistry**

PC3 cells (WT, CD146 KO) were cultured on Poly-L-ornithine (Sigma-Aldrich, St. Louis, MO, USA; P3655) coated 15 mm round cover glass in media containing 10% FBS at 37°C and 5% CO_2_ overnight. Cells were fixed in 4% paraformaldehyde for 10 min at room temperature, washed three times with PBS, permeabilization in 0.1% triton-X100 for 10 min at room temperature, washed with PBS three times, blocked in 3% BSA at room temperature for 2 h and incubated with primary antibodies at 4°C for overnight. The cells were washed three times with PBS and stained with secondary antibodies at room temperature for 1 h and mounted with Aqua Poly/Mount solution (Polyscience, Niles, IL, USA; 18606) at room temperature. The cells were imaged using IX83 microscope equipped with an Olympus x60 objective lens (oil, NA 1.30), fluorescent lamp (Olympus, Tokyo, Japan), stage controller (LEP), and CCD camera (ANDOR, Belfast, UK). Images were analyzed using Metamorph software.

**Cell viability assay**

Cell viability was assessed by the 3-(4,5-dimethylthiazol-2-yl)-2,5-diphenyltetrazolium bromide (MTT) assay. PC3 cells (3 x 10^4^ cells/well in 96-well plate) were incubated for 24, 48, or 72 h. At the indicated time, MTT was added at a final concentration of 0.45 mg/ml. After 1–4 h incubation at 37°C, the medium was then aspirated and dimethyl sulfoxide was added for formazan solubilization. The absorbance was measured at a wavelength of 570 nm by using a fluorescence plate reader (Spectra MAX Pro 5, Molecular Devices, San Jose, CA, USA). Cell viability was expressed according to the following formula:

Cell viability (% of MOI 0) = $\frac{\left( Abs sample \right)-(Abs blank)}{\left( Abs MOI 0 \right)-(Abs blank)}*100$

**Generating knock-out cell lines**

PC3 CD146 knockout cells were made with a CRISPR-Cas9 system. Guide RNA sequences for human CD146 (sense 5’-GTGAAGCGTCAGGGCACCCCCGG-3’ and antisense 5’-CCGGGGGTGCCCTGACGCTTCAC-3’) were inserted into the pRGEN vector and pRGEN-reporter (ToolGen). PC3 cells were transfected with pRGEN-CD146, pRGEN-Cas9 and pRGEN-report using Lipofectamine 3000 (Invitrogen), according to the manufacturer’s instructions. After two days, transfected cells were selected with 300ug/mL hygromycin (PhytoTechnology Laboratories, ACR0397045A) for seven days. Cell colonies were isolated after 2 ~ 3 weeks. To check for genome editing, the region surrounding the target site of the guide RNA was amplified using PCR (forward primer: 5’- CCGCACACAGCTGGTCAACGTGGCCA -3’ and reverse primer: 5’- GTGGATCTTGGTCTTGTTCACTTGC -3’). PCR products were subsequently purified via agarose gel extraction and then sequenced. Sequencing of PCR fragments from the *CD146* KO PC3 cells revealed 1 base-pair deletion confirming successful *CD146* KO. Protein expression was confirmed using western blot analysis.

**Scratch Wound Healing**

PC-3 cells were seeded on 6-well plate. When 90% conﬂuence was reached, the culture medium was removed and replaced with a medium without FBS to inhibit cell proliferation. The monolayer was gently scratched across the center of the well with a sterile pipette tip (Ø = 0.1 mm). The scratch injured cells were washed with a medium without FBS and were incubated at 37°C and 5% for 3 days (until wound closure). We calculated the change in a cell-free area, measuring the leading edge. Three independent experiments were conducted, and four points of the wounded area were analyzed for each replicate. The percentage of the cell-free area was calculated referring to images at T0, for each sample.

PC3 cells were seeded in a 96-well Essen ImageLock plate (Essen BioScience) and were grown to confluence in a CO2 humidified incubator. After 24 h, the scratch was made using the 96-pin WoundMaker (Essen BioScience). Wound images were taken every 2h for 72 h, and the data were analyzed by the integrated metric Relative Wound Density part of the live content cell imaging system Incucyte HD (Essen BioScience).

**Clonogenic assay**

PC3 cells were seeded (250 cells/well) in 12-well plate. The cells were cultured medium with FBS for 7 days. At the end point, culture medium was removed from the wells and cells were fixed with 100% methanol and stained with Crystal Violet. After 10 min, the Crystal Violet solution is discarded and wells were washed with deionized water until the wells are clean enough to count the colonies. Colonies were visualized under a brightfield microscope (In Cell Analyzer 2500; Cytiva, Marlborough, MA, USA). The number of colonies or colony area were quantified through the Image J.

**Immunohistochemistry (IHC)**
Human prostate cancer- normal tissue array samples (CA4) were purchased from SUPER BIO CHIPS (Seoul, South Korea). Immunohistochemistry was performed on the tissue array samples using standard protocols. Briefly, tissue sections were deparaffinized, rehydrated, and subjected to antigen retrieval by heating in a Tris-EDTA buffer (pH 9.0) for 25 minutes. Endogenous peroxidase activity was blocked with 2% BSA + 10% goat serum in 0.3% PBST for 30 minutes. The sections were then incubated with primary antibodies specific for CD146 (dilution 1:250) overnight at 4°C. After washing, sections were incubated with a biotinylated secondary antibody, followed by streptavidin-HRP, and visualized using DAB (3,3'-diaminobenzidine) as the chromogen. Hematoxylin was used as a counterstain. The stained slides were then imaged using a light microscope.

**Statistical analysis**

Experiments were repeated at least three times with consistent results. Unless otherwise stated, data are expressed as the mean ± SD. ANOVA was used to compare experimental groups to control values. Comparisons between multiple groups were performed using a student T-test. Results were statistically significant at *p < 0.05.

**References**

1. Marques RB, van Weerden WM, Erkens-Schulze S, et al. The human PC346 xenograft and cell line panel: a model system for prostate cancer progression. *European urology.* 2006;49(2):245-257.

2. Duijvesz D, Burnum-Johnson KE, Gritsenko MA, et al. Proteomic profiling of exosomes leads to the identification of novel biomarkers for prostate cancer. *PloS one.* 2013;8(12):e82589.

3. Hegmans JP, Bard MP, Hemmes A, et al. Proteomic analysis of exosomes secreted by human mesothelioma cells. *The American journal of pathology.* 2004;164(5):1807-1815.

**Supplementary Figures**

**Fig S1. Flow chart for the exosome purification procedure**

**Conditional media**

**Supernatant**

**Supernatant**

**pellets**

**Resuspend pelleted exosomes in PBS**

**( using as an Exosome Enriched Medium)**

**2,000 rpm, 5min, 4℃**

**3,000 rpm, 30min, 4℃**

**30,000 rpm, 60min, 4℃**

**SW 41 Ti**

**30,000 rpm, 60min, 4℃**

**Wash in PBS**

Flow chart for the exosome purification procedure based on differential ultracentrifugation. The speed and the length of each centrifugation steps are indicated at the central line of the arrows. After each the first step, pellet is discarded, and the supernatant is kept for the next step. In contrast, after the two 100,000 × g centrifugations, pellets are kept, and the supernatants are discarded

**Fig S2. Silver–stained SDS polyacrylamide gel after separation of 20 µg of total exosomes (Exo) from PC-3 and LNCaP cells**

**
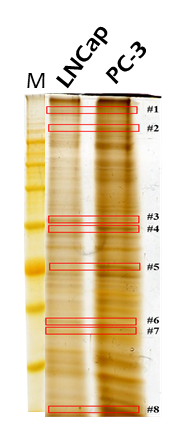
**

**Fig S3.** LC-MS/MS preparation.

**
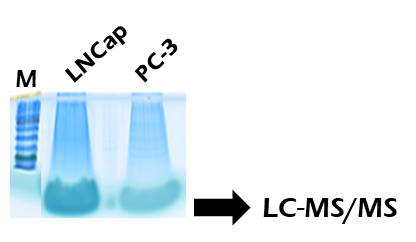
**

**Fig S4. Comparative analysis of CD146 expression in prostate cancer tissues stratified by Gleason scores of 6 and 9.**


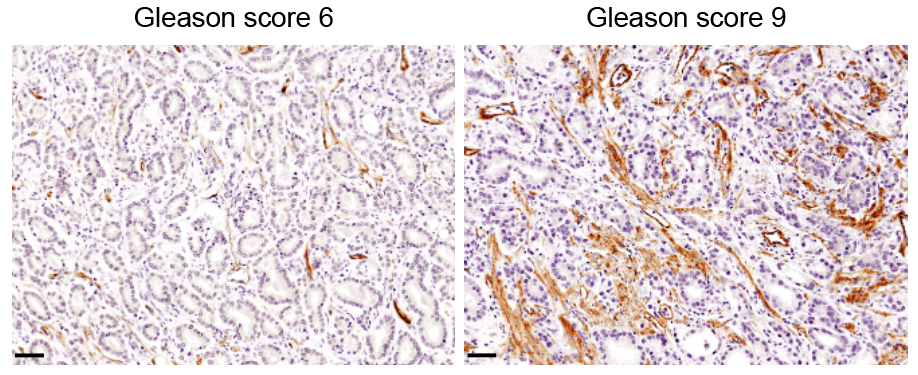

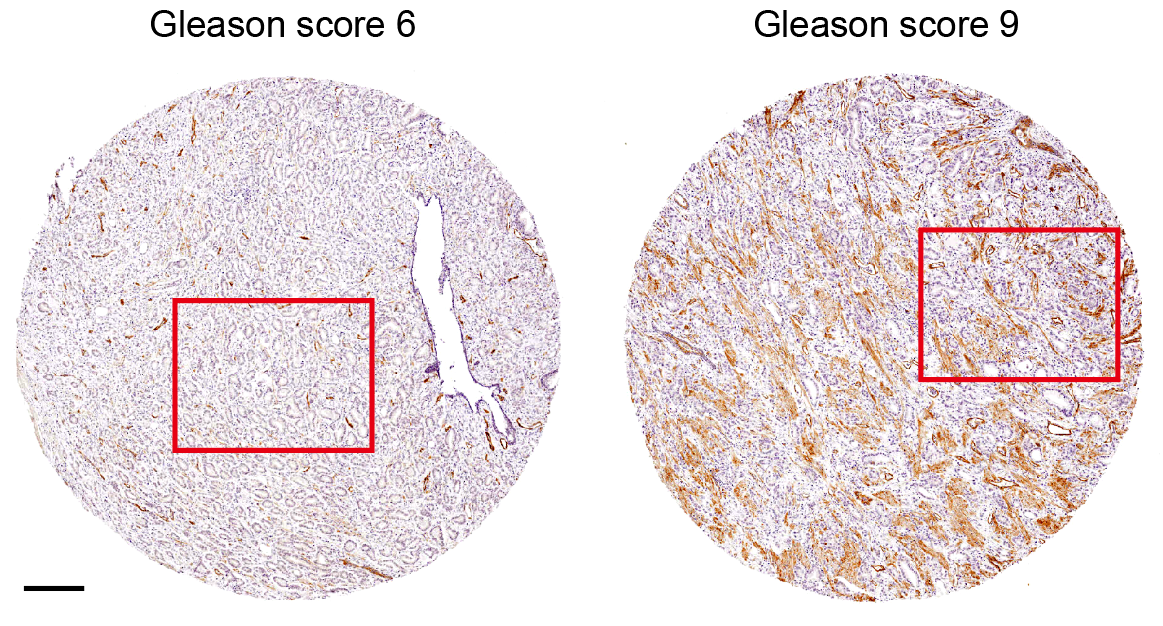

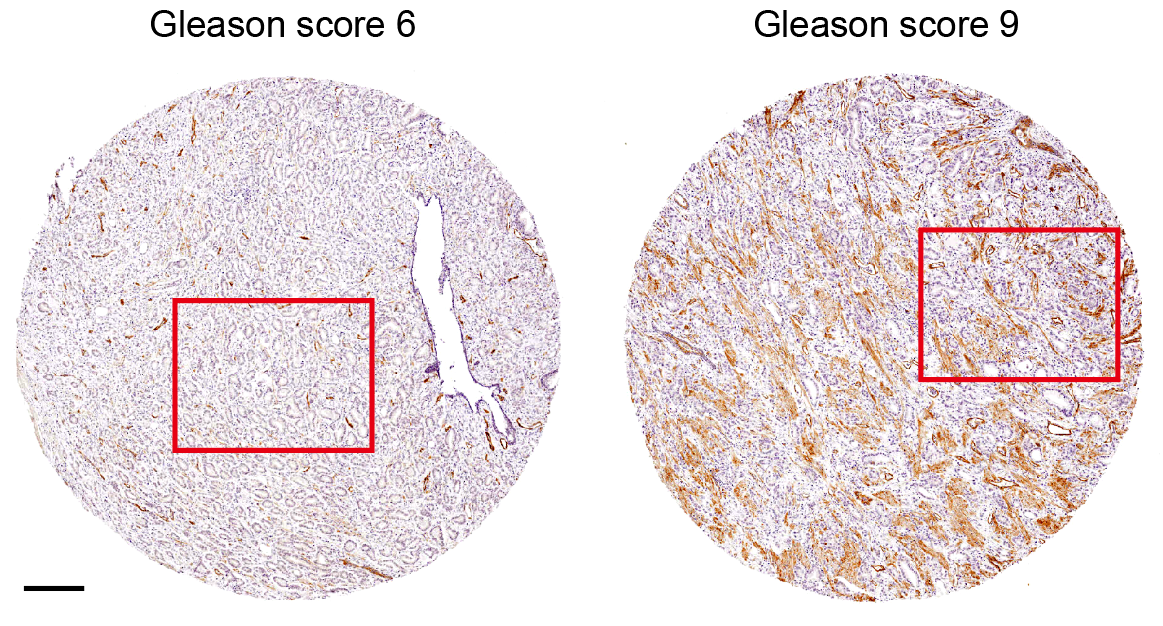


**Supplementary Table 1.** Summary for LC-MS/MS data analysis of PC-3 and LNCaP exosomes using scaffold **Software**

**
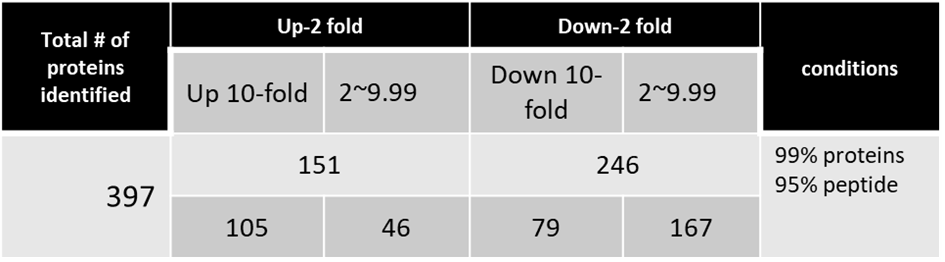
**

**Supplementary Table 2**. 20 up-regulated genes identified in proteomic analyses


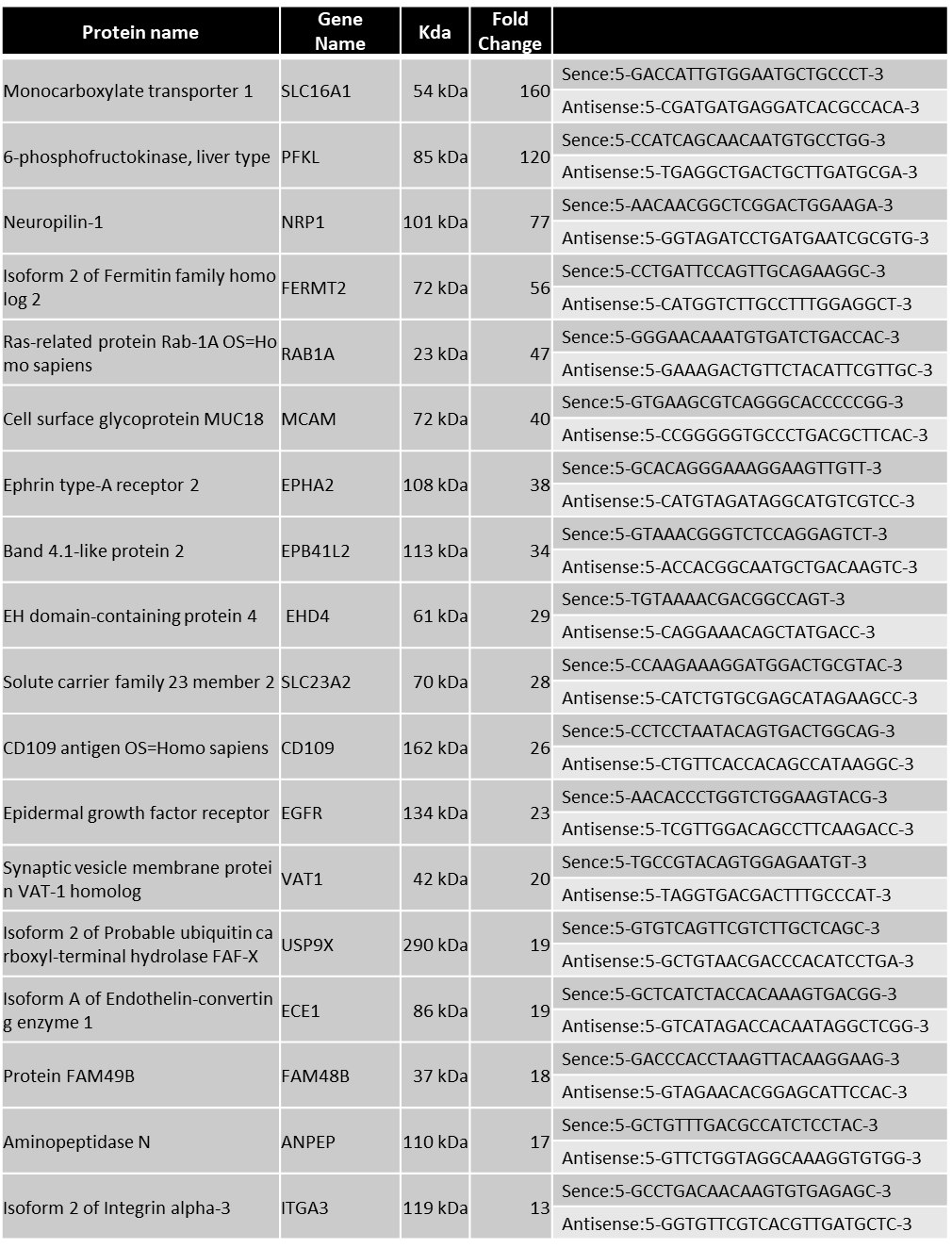


List of the exosomal proteins suggested as potential prostate cancer biomarkers. 20 up-regulated proteins are considered for validation due to their relative high abundance. cDNA Primers encoding the proteins are included.
