# Supplementary material for: Proteomic profiling of exosomes leads to the identification of a candidate biomarker for prostate cancer progression
Source: Genes Dis. 2024 Nov 12;12(4):101463. doi: 10.1016/j.gendis.2024.101463 (PMC11995069; doi:10.1016/j.gendis.2024.101463)
Supplement: Multimedia component 2 [file mmc2.pptx]

## Slide 1
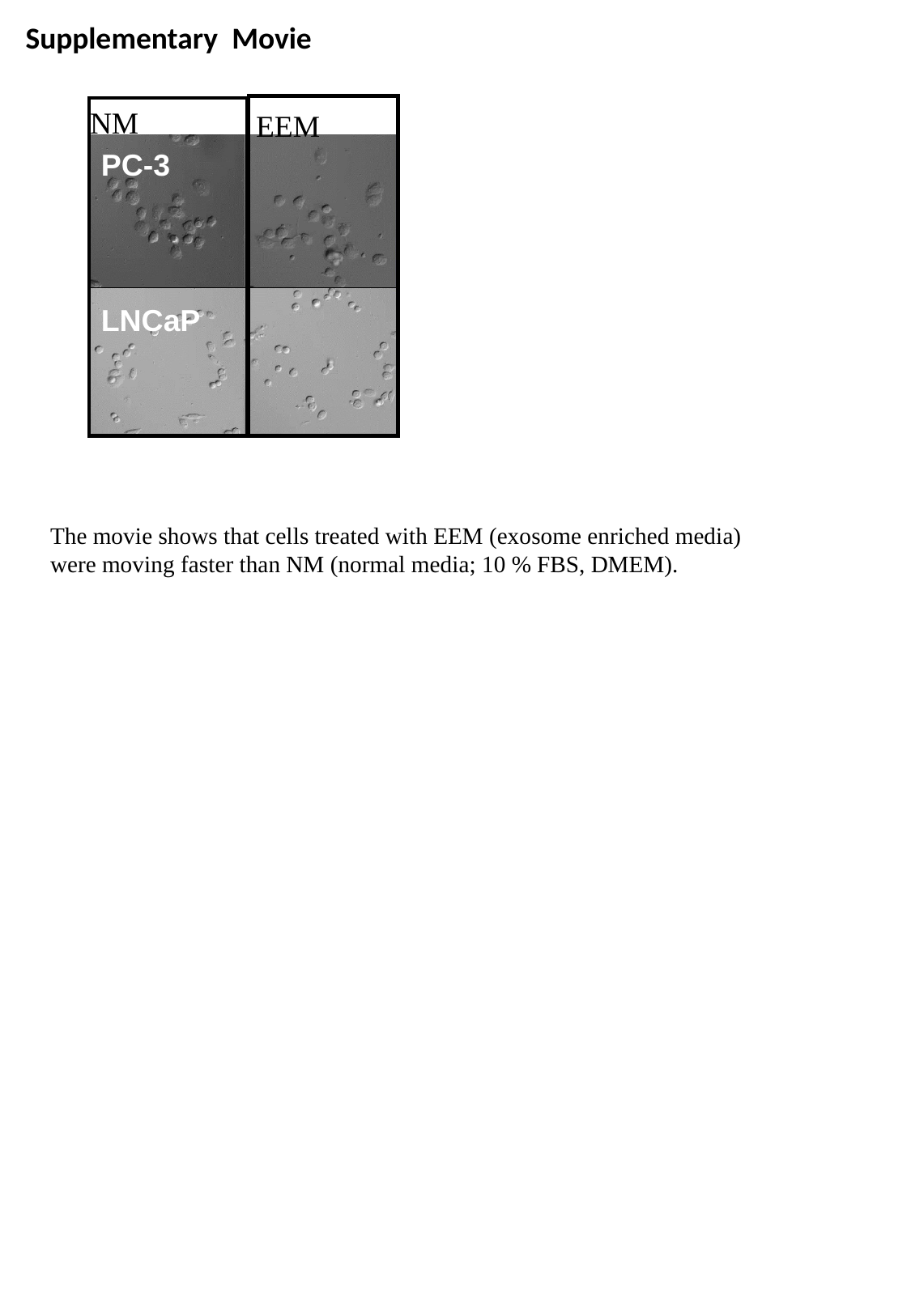

Supplementary Movie
NM
EEM
PC-3
LNCaP
The movie shows that cells treated with EEM (exosome enriched media) were moving faster than NM (normal media; 10 % FBS, DMEM).
